# A Geometric Framework for Predicting Donor Area Thinning After Follicular Unit Excision

**DOI:** 10.1111/jocd.71156

**Published:** 2026-09-01

**Authors:** Georgios Zontos, Aditya K. Gupta, Vasiliki Economopoulos, Paul T. Rose, Mesbah Talukder

**Affiliations:** ^1^ Department of Medicine University of Patras Patras Greece; ^2^ Zontos Hair and Skin Clinic Glyfada Greece; ^3^ Division of Dermatology, Temerty Faculty of Medicine University of Toronto Toronto Ontario Canada; ^4^ Mediprobe Research Inc. London Ontario Canada; ^5^ Golden State Dermatology Pleasant Hill California USA; ^6^ School of Pharmacy BRAC University Dhaka Bangladesh

## Abstract

**Background:**

Safe donor harvesting in follicular unit excision (FUE) is traditionally estimated using donor density alone, despite substantial variation in donor appearance among patients with similar densities.

**Objective:**

To develop a geometric framework for estimating donor coverage and predicting visible donor thinning based on individual hair characteristics.

**Methods:**

A mathematical model was developed incorporating hair shaft diameter, hair length, exit angle, and donor density. The projected scalp coverage of each hair shaft was approximated geometrically, allowing calculation of minimum residual hair density and maximum safe harvestable graft number. Clinical examples were used to illustrate model behavior.

**Results:**

The model demonstrated that safe donor capacity is highly sensitive to variations in hair caliber, length, and exit angle. Patients with identical donor densities showed markedly different predicted harvesting limits based on hair geometry alone. Increased hair caliber and hair length substantially improved coverage, whereas steeper exit angles reduced donor yield.

**Conclusion:**

This framework provides a quantitative, geometry‐based approach to donor assessment and may assist individualized donor planning and reduction of overharvesting risk.

## 1 Introduction

One of the most significant challenges in follicular unit excision (FUE) surgery is determining how much of the donor area can be safely harvested without producing visible thinning. In FUE overharvesting remains a common complication [1, 2]. Visible donor depletion can occur even when the surgeon follows seemingly safe extraction limits, because the optical appearance of density is affected by more than graft count alone [2].

Traditional methods of estimating donor capacity rely mainly on hair density per square centimeter [3], which is an incomplete measure of true coverage. In reality, the visual fullness of hair depends on multiple physical characteristics: (1) the diameter of the hair shaft, (2) the length of the hair, and (3) the angle at which each hair shaft exits the scalp surface.

The present study introduces a model that integrates these factors into a simple mathematical equation. This model assists surgeons in predicting when the donor area will appear thin, how much hair can be safely extracted, and how donor visibility changes with varying hair characteristics.

## 2 Previous Approach Versus Current Approach

Previous approaches have attempted to quantify visual coverage through indices such as the Hair Diameter Index (HDI) and the Coefficient of Coverage (CoC) [4]. HDI is defined as the hair shaft diameter in microns multiplied by the density of hair as hairs/cm^2^ and then divided by 100 [5]. The CoC (sometimes also referred to as the hair coverage value) has a similar calculation as the HDI [6]. The CoC is the hair shaft diameter in mm multiplied by the hair density as hairs/cm^2^ [2]. These indices have proved helpful in providing guidance regarding the amount of donor area that can be harvested.

However, the HDI and CoC do not incorporate the exit angle (*θ*) or length (l) of the hair shaft [3, 4, 6]. When a hair shaft emerges from the scalp at an acute angle, it projects over a larger surface area, enhancing optical coverage. Conversely, a larger exit angle (exit angle at a more perpendicular level to the scalp) produces less overlap and may demonstrate more visible thinning, even at the same density. In addition, thinner caliber hair will expose more underlying scalp, limiting the amount of hair that can be removed from the donor area [6]. Similarly, longer hair shafts create larger overlapping projections, enhancing the appearance of coverage—whereas short or trimmed hairs expose more of the underlying scalp.

Until now, the critical value at which the donor density appears thin has been based on physician experience and is a subjective assessment. This study introduces a more integrated mathematical approach to determine what level of coverage is needed in the donor area to prevent visible thinning, based on both density and the geometric characteristics of the patient's hair within the donor area. Therefore, it helps determine the safe harvesting limits for each individual patient, enabling better long‐term donor planning and post‐harvest assessment.

## 3 Material and Methods

**FIGURE 1:**
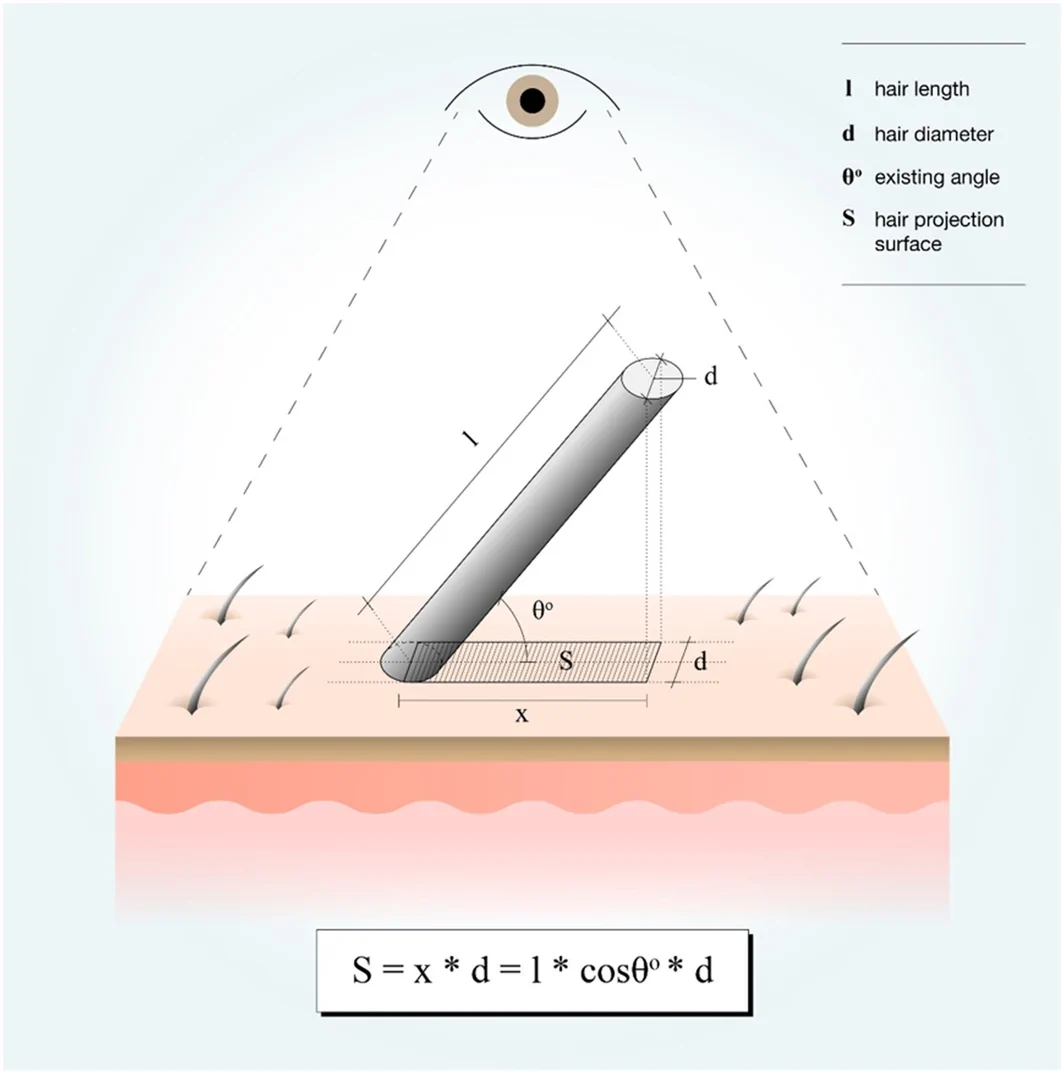
Geometric representation of hair coverage and the effect of exit angle on projected area.

In this model, for simplicity reasons, each hair is represented as a cylindrical structure of length (l) and diameter (d) that exits the scalp at an angle (*θ*) relative to the scalp surface. The visible surface area covered by one hair on the scalp is proportional to its projection on the scalp plane, where a low value for *θ* would create a larger hair projection on the scalp. The projection of one hair on the scalp surface can be approximated by a rectangular area (Figure 1). We can use this geometric formula which is shown in Figure 1 to then determine the total available hair coverage and the amount of hair that can be safely removed before the appearance of thinning in the donor area.

### 3.1 Mathematical Explanation

The visual density of the donor area after follicular unit extraction (FUE) depends not only on hair density, but also on the geometric characteristics of the hair shaft and its orientation relative to the scalp surface.

Model parameters: *d* = hair diameter, *l* = hair length (cm), *θ* = exit angle relative to the scalp surface, *N* = number of hairs, *S*_*d*_ = donor surface area, and density = hairs/cm^2^.

#### 3.1.1 Projected Surface Contribution of a Single Hair

The projected coverage of a single hair shaft is approximated by:

(1) $$S=l\cdot d\cdot \cos\left(\theta \right)$$

representing the geometric projection of a cylindrical shaft inclined at angle *θ*.

#### 3.1.2 Total Visual Coverage of the Donor Area

For *N* hairs, total projected coverage becomes:

(2) $$S_{c}=N\cdot l\cdot d\cdot \cos\left(\theta \right)$$

#### 3.1.3 Minimum Hair Number Required to Prevent Visible Thinning

To maintain visual coverage:

(3) $$S_{c}\geq S_{d}$$

therefore:

(4) $$N_{\min}=S_{d}/\left(l\cdot d\cdot \cos\left(\theta \right)\right)$$

#### 3.1.4 Maximum Safe Harvestable Hair Number

The total donor hair number is:

(5) $$N_{\text{donor}}=S_{d}\cdot \text{Density}$$

Thus, the maximum safely harvestable hair number is:

(6) $$N_{\text{harvest}}=N_{\text{donor}}–N_{\min}$$

or:

(7) $$N_{\text{harvest}}=S_{d}\cdot \text{Density}-S_{d}/\left(l\cdot d\cdot \cos\left(\theta \right)\right)$$

This model suggests that patients with identical donor densities may exhibit different safe extraction limits depending on hair caliber (*d*), hair length (*l*), and exit angle (*θ*), supporting a more individualized approach to donor management.

**FIGURE 2:**
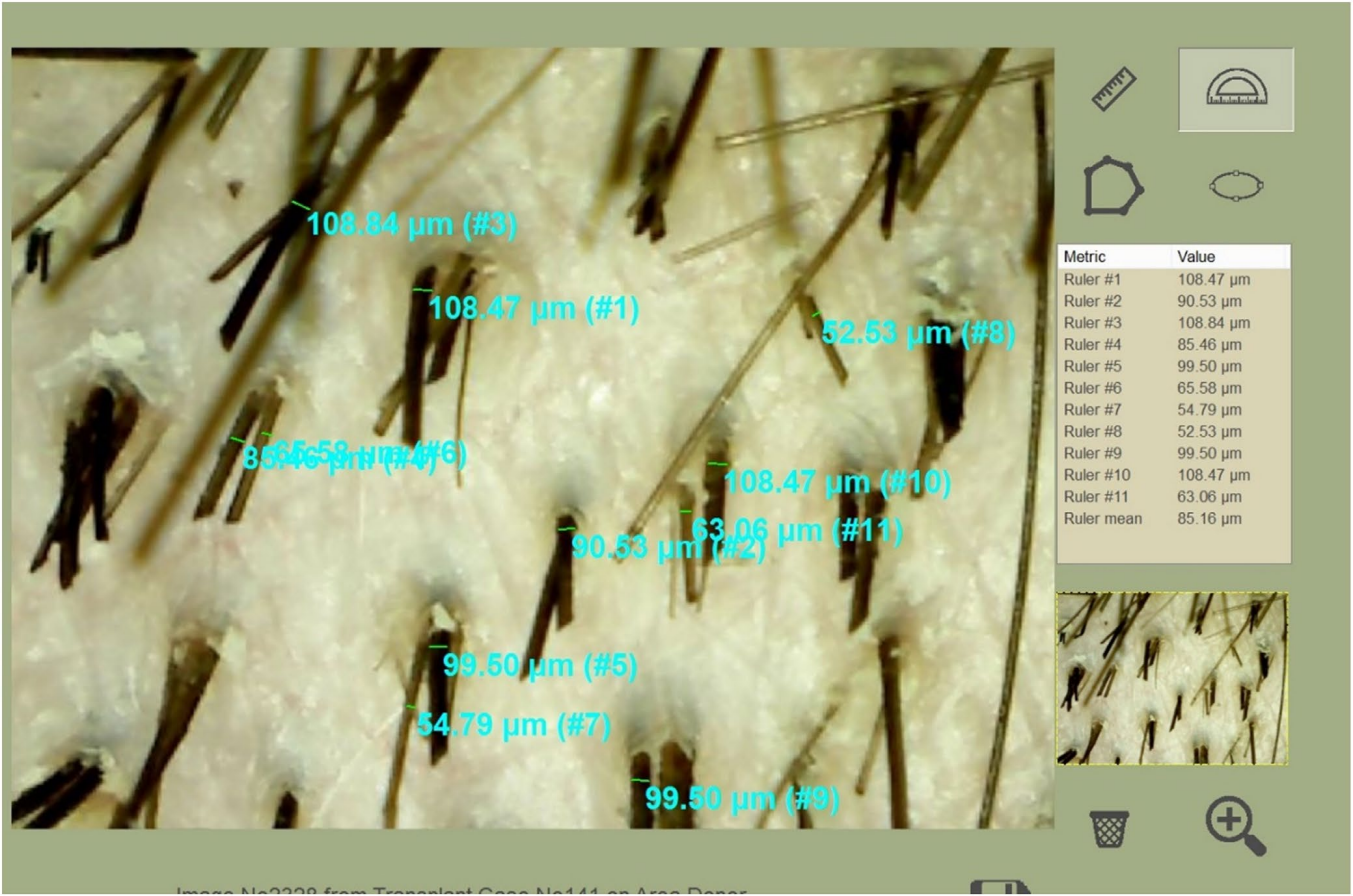
Automatic calculation of the average hair caliber.

**FIGURE 3:**
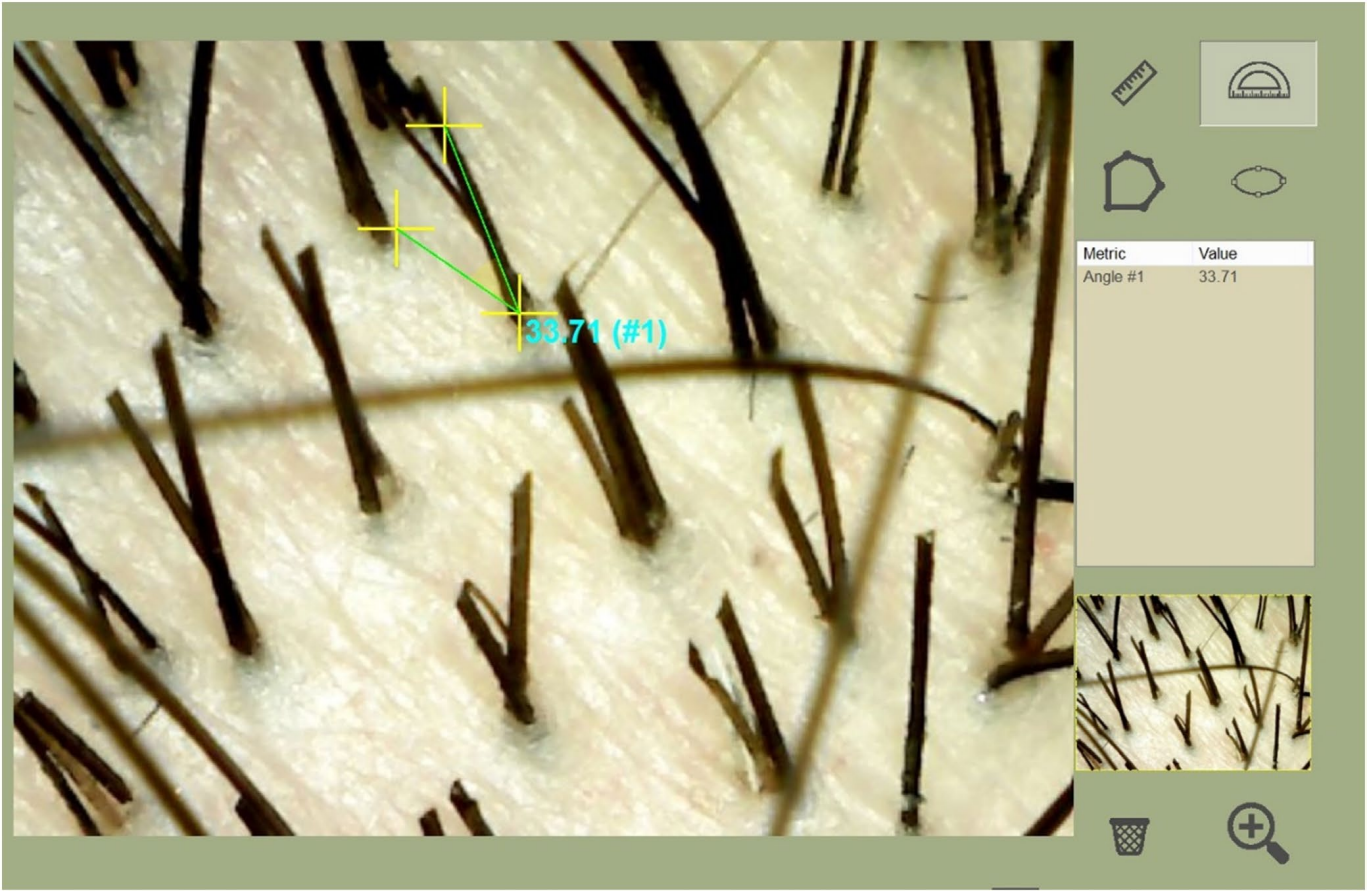
Automatic measurement of exit angle, using digital trichoscopy.

**FIGURE 4:**
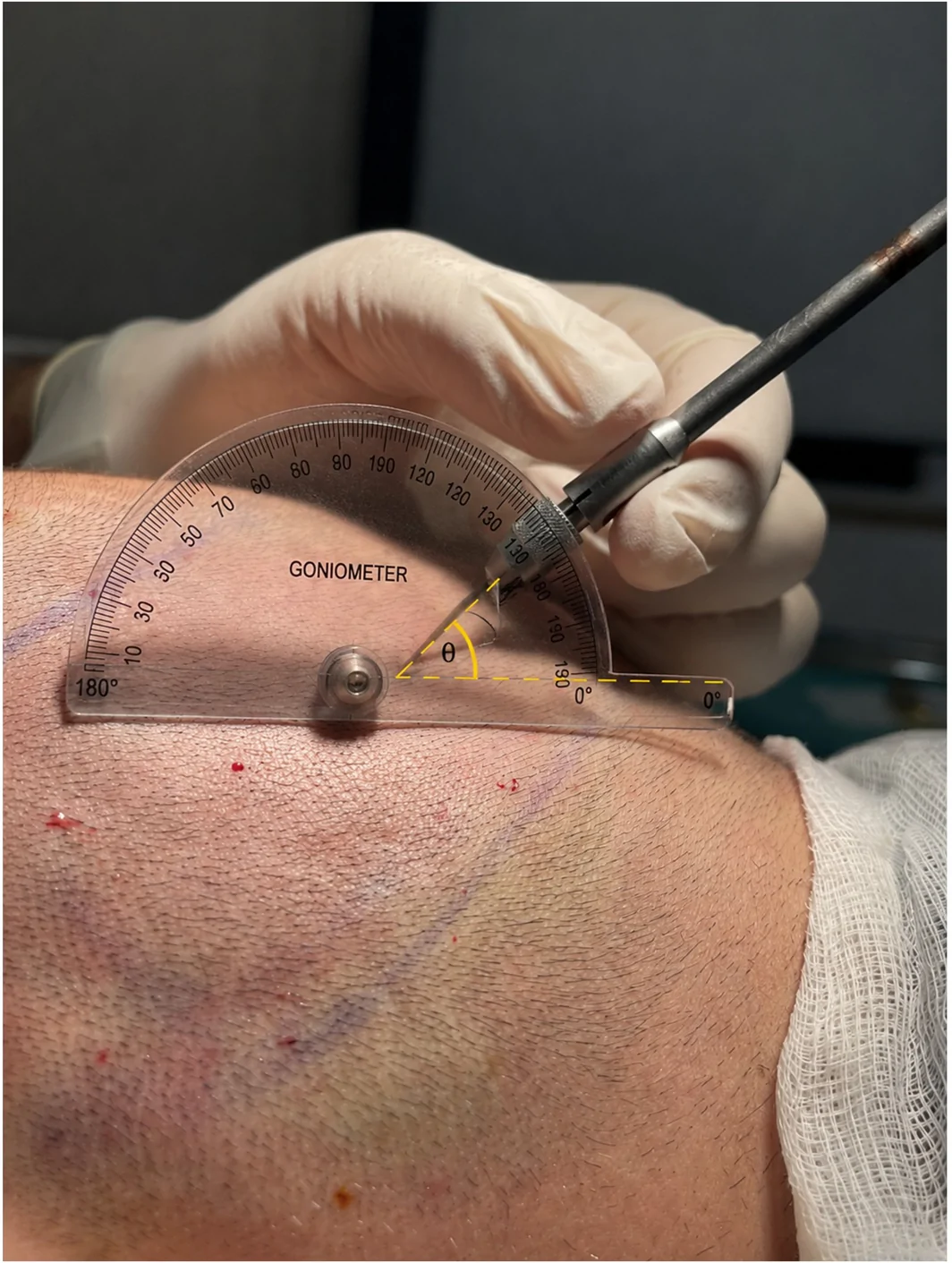
Measurement of hair exit angle using a goniometer.

**FIGURE 5:**
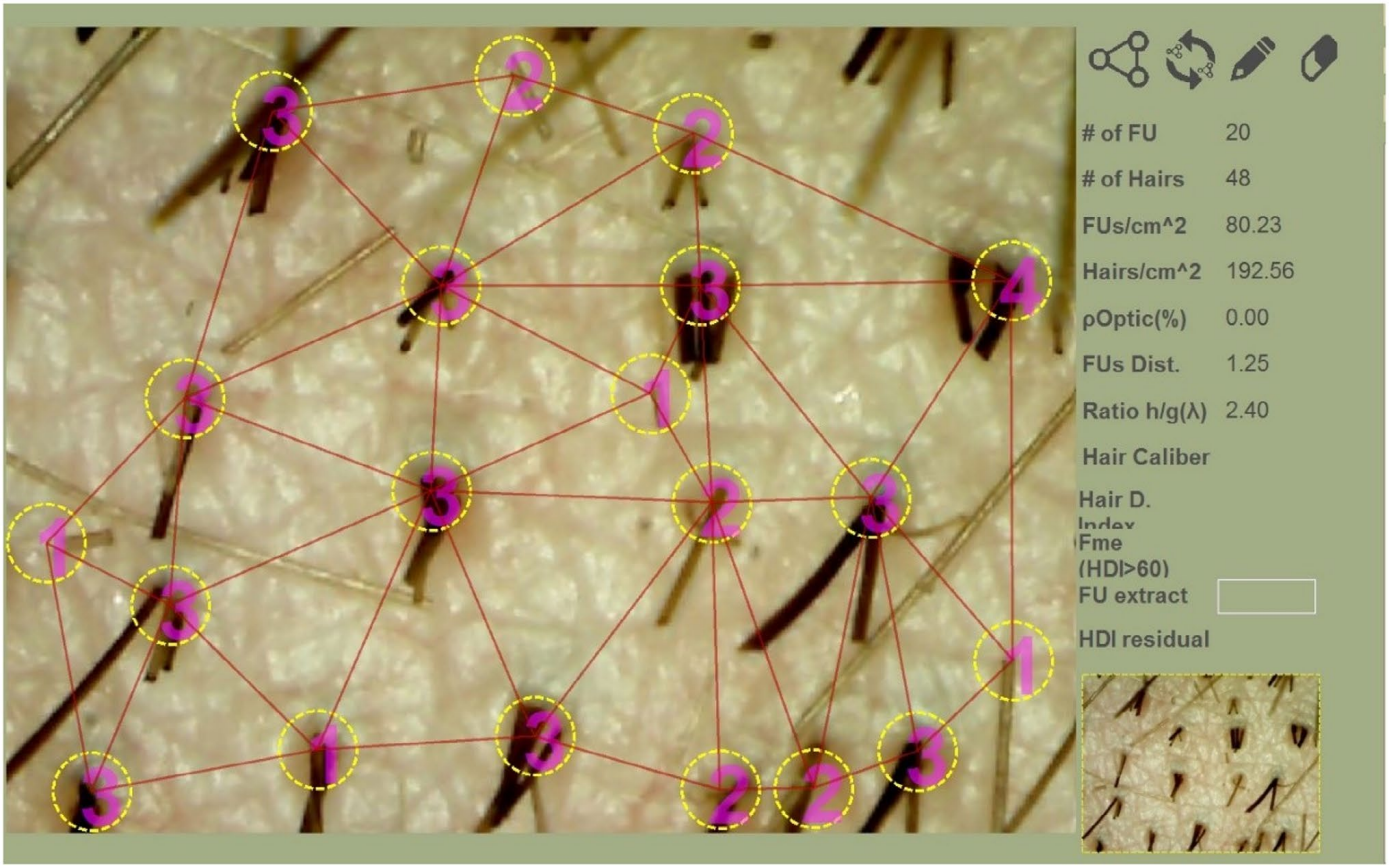
Follicular unit density (FU/cm^2^), hair density (hairs/cm^2^), and the average number of hairs per follicular units using image processing.

Hair shaft diameter and hair exit angle can be measured using calibrated digital trichoscopy with integrated image‐analysis software, developed by the first author and routinely employed in clinical practice for hair restoration assessment. Specifically, hair caliber (μm) was determined by direct digital measurement of the transverse diameter of multiple representative hair shafts within the magnified trichoscopic image and the mean value was automatically calculated by the software (Figure 2). Hair exit angle (*θ*) was measured digitally by defining the axis of the emerging hair shaft relative to the scalp surface plane using geometric angle analysis (Figure 3). Alternatively, the exit angle may be estimated clinically by aligning a cylindrical punch parallel to the hair shaft and measuring the angle formed between the punch axis and the scalp surface using a goniometer (Figure 4). Follicular unit density (FU/cm^2^), hair density (hairs/cm^2^), and the average number of hairs per follicular unit were calculated by counting follicular units and individual hairs within a calibrated scalp surface area using an advanced image processing system (Figure 5).

### 3.2 Example Calculations

**FIGURE 6:**
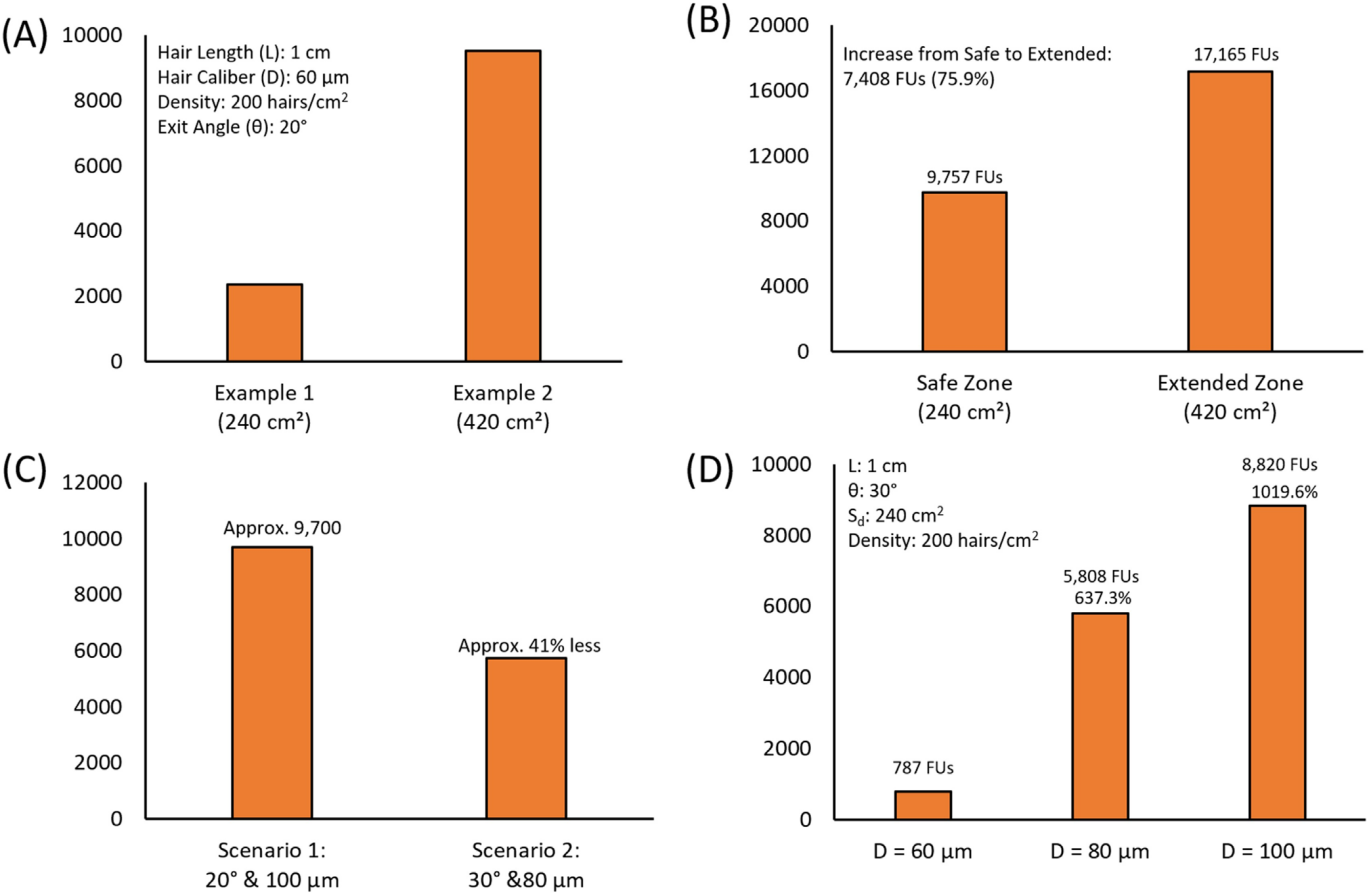
Effect of hair geometry on safe donor yield. (A) Influence of donor surface area with constant hair caliber (60 μm). (B) Effect of increased hair caliber (100 μm) and donor area on graft yield. (C) Relationship between exit angle, hair diameter, and safe graft number. (D) Nonlinear increase in safe donor yield with increasing hair caliber (60–100 μm).

For a donor area of 240 cm^2^ (classical ‘safe’ area), with an average hair diameter of 60 μm, exit angle of 20°, hair length of 1 cm, and a density of 200 hairs/cm^2^ (87 grafts/cm^2^, assuming roughly 2.3 hairs per follicular unit (FU) [7, 8]), the model predicts that there are 5436 hairs (2363 grafts) that can safely be harvested from within the donor area whilst maintaining scalp coverage (Figure 6A).

Based on Equation (7) we can find: *N*_harvest_ = *N*_donor area_ − *N*_min_.

*S*_*d*_ = 240 cm^2^, *l* = 1 cm, *d* = 60 μm (0.006 cm); *density* = 200 hairs/cm^2^, *θ* = 20°.

*N*_harvest_ = [*S*_*d*_ * hair density] − *S*_*d*_/(l*d*cos*θ*).

*N*_harvest_ = (240 cm^2^) * (200 hairs/cm^2^) − (240 cm^2^)/[(1 cm) * (0.006 cm) * cos(20°)] = (48 000 hairs) − (42 564 hairs) = 5436 hairs (2363 grafts) available for harvest and still maintain scalp coverage.

#### 3.2.1 Effect of Donor Area Expansion on Donor Yield

Expanding the donor zone from 240 to 420 cm^2^ increased the predicted safely harvestable number to 9513 hairs (4135 grafts) using the same geometric parameters (Figure 6A).

#### 3.2.2 Effect of Increased Hair Caliber on Donor Yield

In a typical occipital donor area of 240 cm^2^, increasing hair caliber from 60 to 100 μm (*θ* = 20° = *l* = 1 cm) reduced the minimum density required for visual coverage to 25 559 hairs (≈106 hairs/cm^2^). With an initial donor count of 48 000 hairs (20 869 grafts), the model predicted 22 441 hairs (9756 grafts) safely available for harvest (assuming 2.3 hairs/FU) [7] (Figure 6B).

#### 3.2.3 Effect of Donor Area Expansion Outside the Safe Donor Area and Increased Hair Caliber

Expanding the donor zone outside the classical safe donor area (240–420 cm^2^) and increasing hair caliber from 60 to 100 μm increased the total donor count to 84 000 hairs (36 521 grafts), with 39 480 hairs (17 165 grafts) safely available for harvest (Figure 6B).

These calculations illustrate how selected patients with favorable hair geometry and extended donor utilization may tolerate substantially larger cumulative graft numbers than predicted by conventional density‐based approaches.

#### 3.2.4 Effect of Hair Exit Angle and Caliber on Donor Yield

The model demonstrates high sensitivity to variations in hair exit angle and shaft diameter (Figure 6C). Increasing the exit angle from 20° to 30° reduces projected scalp coverage through the cosine‐dependent component of the equation, while reducing hair diameter from 100 to 80 μm further decreases coverage. Together, these changes increase the minimum residual hair count required to maintain visual coverage from 25 559 to 34 642 hairs, corresponding to a 41% reduction in graft capacity. Consequently, patients with identical donor densities may exhibit markedly different safe extraction limits based solely on differences in hair geometry.

#### 3.2.5 Effect of Hair Caliber on Donor Yield

To evaluate the effect of hair caliber on donor safety, a standardized donor model was analyzed using fixed parameters: hair length *l* = 1 cm, exit angle *θ* = 30°, donor surface area *S*_*d*_ = 240 cm^2^, and density = 200 hairs/cm^2^ (Figure 6D). Three hair diameters were compared: 60, 80, and 100 μm.

Under identical conditions, the model predicted 1812 safely harvestable hairs (≈788 FUs) at 60 μm, 13 359 hairs (≈5808 FUs) at 80 μm, and 20 287 hairs (≈8821 FUs) at 100 μm. These findings demonstrate the strong nonlinear effect of hair caliber on donor capacity. Even with identical density, exit angle, and donor surface area, patients with finer hair reach visible donor depletion at substantially lower extraction levels than patients with thicker hair.

#### 3.2.6 Effect of Hair Exit Angle and Caliber on Donor Yield

**FIGURE 7:**
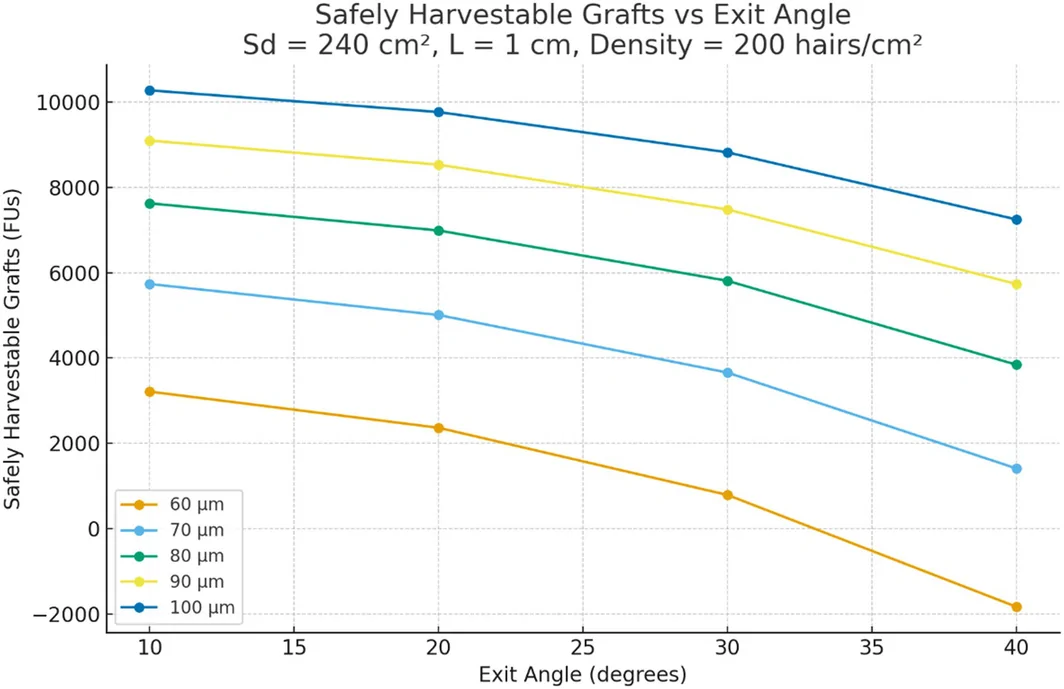
Relationship between exit angle and safe graft yield across different diameters.

Figure 7 illustrates the relationship between hair exit angle (10°–40°) and safely harvestable grafts for five different hair calibers (60–100 μm) using a standardized donor model (S_d_ = 240 cm², l = 1 cm, density = 200 hairs/cm^2^; 87 grafts/cm^2^ assuming 2.3 hairs/FU). Increasing the exit angle produced a cosine‐dependent nonlinear reduction in graft yield across all diameters, whereas increasing hair caliber shifted the curves upward, confirming hair diameter as a major determinant of donor safety. The combination of fine hair and steep exit angle produced the lowest safe harvesting capacity.

### 3.2 Hair Curvature Considerations

Although hair curvature may influence donor and recipient area coverage, a detailed mathematical analysis of curly and wavy hair falls beyond the scope and primary objectives of the present study. Unlike straight hair, curved hair shafts follow complex three‐dimensional trajectories that are difficult to model accurately using simple geometric assumptions. Furthermore, the visual appearance of hair density in curly hair is affected not only by geometry but also by optical phenomena such as fiber overlap, spatial dispersion, self‐shadowing, and light scattering. A comprehensive mathematical description of these interactions would require substantially more advanced geometric and computational modeling. Nevertheless, hair curvature should be considered when interpreting donor appearance and estimating safe harvesting limits in selected patients. For completeness, the following conceptual considerations are provided.

The present geometric model was developed primarily for straight hair shafts. Curly and wavy hair introduces additional complexity because the hair shaft follows a non‐linear path and may create greater apparent coverage through fiber overlap, spatial dispersion, self‐shadowing, and light‐scattering effects. Consequently, individuals with curly hair often appear to have greater visual hair density than would be predicted by linear geometric projection alone. This observation may partially explain why some patients with curly or wavy hair can tolerate higher extraction densities before visible donor thinning becomes apparent. However, the quantitative contribution of hair curvature to donor coverage remains incompletely understood and requires further clinical investigation.

### 3.3 Clinical Application of the Geometric Model

The model was compared with real surgical data from clinical FUE procedures.

**FIGURE 8:**
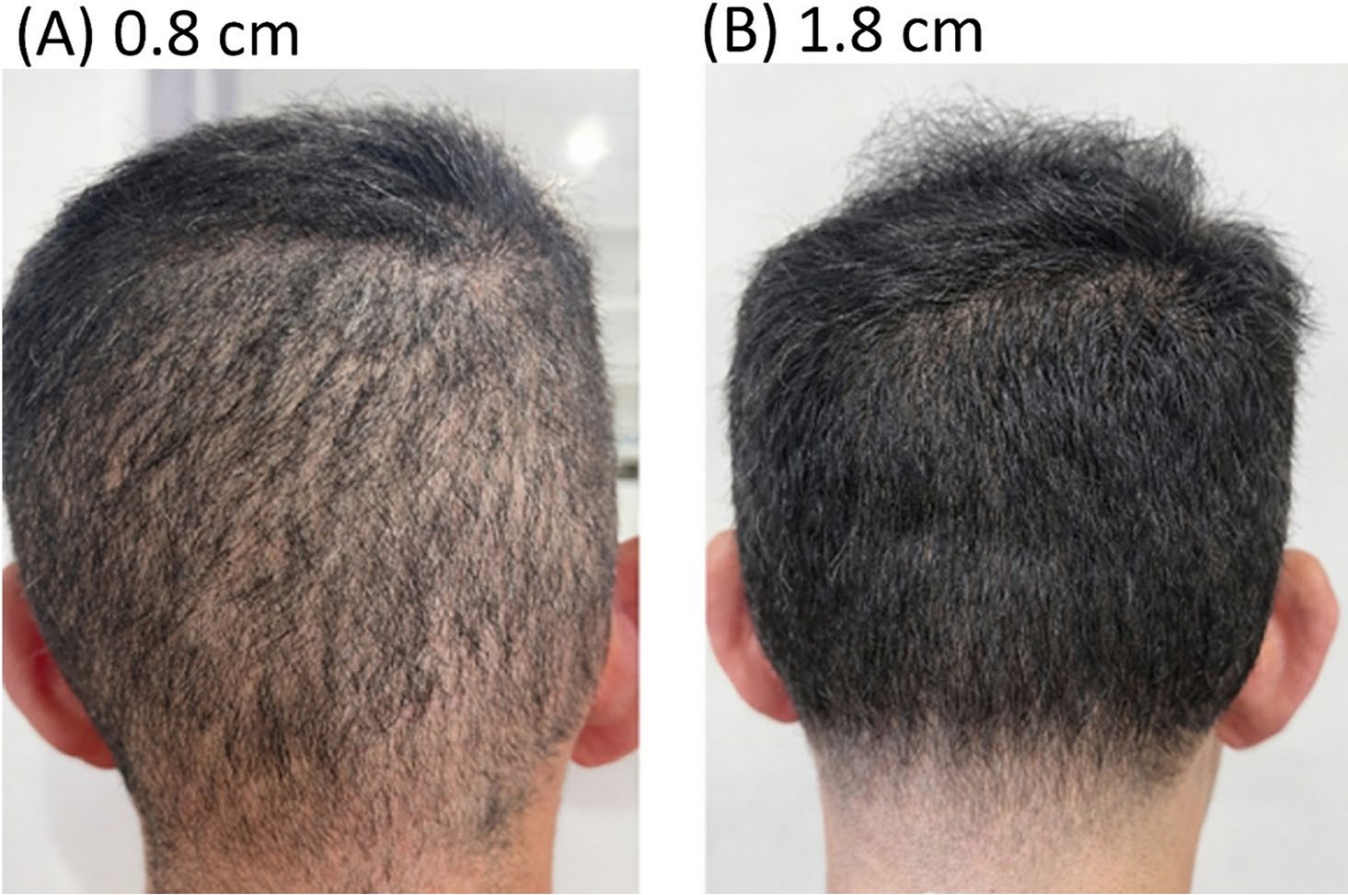
Patient A with two different hair lengths. (A) Short donor hair (~0.8 cm) showing visible thinning below the geometric coverage threshold. (B) Longer donor hair (~1.8 cm) restoring visual density despite identical residual density and extraction pattern.

Specifically, in Figure 8, we highlighted a patient (Patient A) who underwent an FUE procedure. The patient's hair was 80 μm in diameter, and the exit angle was 35° and the residual hair density is 140 hairs/cm^2^. In panel A, the hair is cut to 0.8 cm in length where the minimum density post FUE is approximately 191 hair/cm^2^. Visible thinning can be seen at this hair length. In panel B, the patient's hair is now 1.8 cm in length where the minimum density required post FUE is now approximately 85 hairs/cm^2^. In this condition, the patient's hair appears visually dense, highlighting the effect of the hair's geometry on donor area appearance.

**FIGURE 9:**
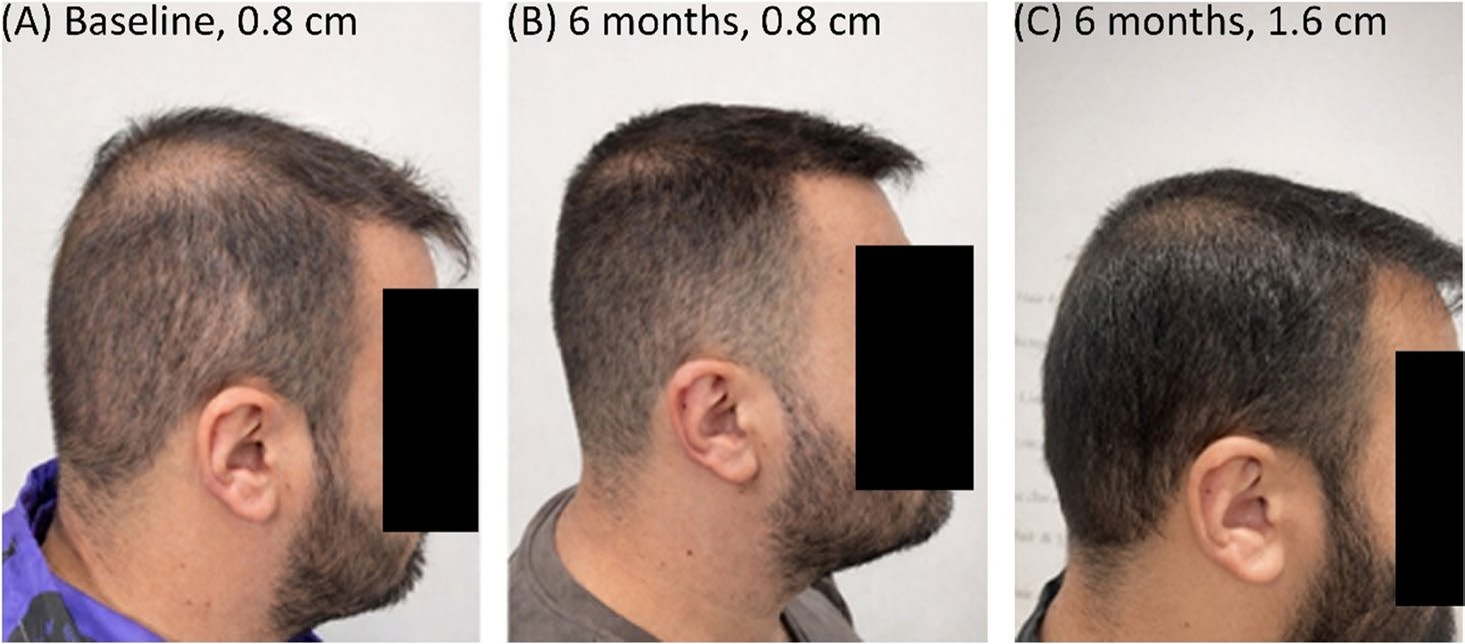
Patient B treated with oral minoxidil demonstrating changes in donor coverage. (A) Baseline appearance with 0.8 cm hair length and visible thinning. (B) Improved coverage after 6 months of oral minoxidil at the same hair length (0.8 cm). (C) Further improvement in visual density after increasing hair length to 1.6 cm.

In a second example (Figure 9), we present a clinical case (Patient B) which was analyzed to evaluate changes in donor appearance using our model. Panel A represents the donor area three years after an FUE procedure, demonstrating visible thinning secondary to donor overharvesting. The patient subsequently received oral minoxidil 5 mg daily, and follow‐up photographs (panels B and C) were obtained six months after initiation of therapy.

The geometric and clinical characteristics of Patient B in Figure 9 are listed in Table 1.

**TABLE 1:** Clinical and geometric parameters of Patients A and B.

Clinical context	Patient A		Patient B		
	Shorter hair	Longer hair	3 years post‐FUE overharvesting	After 6 months oral minoxidil 5 mg/day	Same patient, longer hair
Hair length (l)	8 mm (0.8 cm)	18 mm (1.8 cm)	8 mm (0.8 cm)	8 mm (0.8 cm)	1.6 cm
Exit angle (*θ*)	35° (cos*θ* ≈ 0.82)	35° (cos*θ* ≈ 0.82)	20° (cos*θ* ≈ 0.94)	20° (cos*θ* ≈ 0.94)	20° (cos*θ* ≈ 0.94)
Hair shaft diameter (*d*)	80 μm	80 μm	60 μm	80 μm	80 μm
Estimated density (actual)	140 hairs/cm^2^	140 hairs/cm^2^	150 hairs/cm^2^	162 hairs/cm^2^	162 hairs/cm^2^
Calculated minimum density	191 hairs/cm^2^	85 hairs/cm^2^	≈222 hairs/cm^2^	≈166 hairs/cm^2^	≈83 hairs/cm^2^
Geometric position	Below threshold	Well above threshold	Well below threshold	Near threshold (slightly below)	Well above threshold

At baseline (Figure 9A), the donor area demonstrated visible thinning, as the estimated actual density (~150 hairs/cm^2^) was substantially below the calculated geometric coverage threshold (~222 hairs/cm^2^). After 6 months of oral minoxidil therapy (Figure 9B), hair shaft diameter increased from approximately 60 to 80 μm, with a modest density increase (150–162 hairs/cm^2^). This reduced the calculated coverage threshold to ~166 hairs/cm^2^ and improved the visual appearance of the donor area despite minimal density change.

When hair length increased to 1.6 cm (Figure 9C), while density and diameter remained unchanged, the calculated minimum density required for coverage decreased further to ~83 hairs/cm^2^, producing a visibly denser donor appearance. Increased hair length likely enhanced coverage not only by increasing geometric projection but also by reducing the effective exit angle through gravitational bending of the hair shaft, thereby increasing projection onto the scalp surface.

Collectively, these findings suggest that donor appearance is determined by the interaction of hair length, diameter, exit angle, and density rather than density alone.

## 4 Discussion

This geometric model provides a structured framework for estimating donor coverage and the risk of visible thinning after FUE. By incorporating hair length, diameter, and exit angle, it extends traditional density‐based assessment and may help explain why patients with similar donor densities can exhibit different visual outcomes.

For simplification, hair shafts are approximated as cylindrical structures. Although hair morphology may be conical or irregular, this approximation is reasonable for shorter hair lengths (≤ 1 cm), where the geometric assumptions remain relatively stable.

The model represents a first‐order approximation of a biologically and optically complex system and several limitations should be considered. Donor density is not fully uniform across the scalp, and local variations in follicular unit composition, spacing, and regional density may influence coverage [2, 8, 9, 10]. Hair shafts are also treated as independent geometric elements, whereas overlap, clustering, and spatial interactions contribute to visual density in vivo.

Moreover, the present model is intended as a first‐order geometric approximation and is expected to provide the greatest predictive accuracy for relatively short hairstyles, where individual hair shafts behave largely independently and geometric projection predominates. As hair length increases, additional factors—including multilayer stacking, hair overlap, draping, gravitational bending, clustering, and other optical interactions—progressively influence perceived donor coverage. Consequently, the predictive accuracy of the current model may decrease for longer hairstyles, where visual density is determined by a combination of geometric and complex optical phenomena beyond simple linear projection. Future refinements incorporating these factors may further improve the predictive capability of the model.

The model also does not explicitly incorporate optical effects such as light scattering, internal shading, hair–skin color contrast, scalp curvature, or regional variation in exit angle and hair geometry, all of which may affect perceived density.

Accuracy is expected to be greater with shorter hair lengths, where overlap and splaying are minimized. As hair length increases, layering and optical interactions become more prominent, potentially reducing predictive precision.

The exit angle (*θ*) may be estimated using digital trichoscopy with image analysis or by direct geometric alignment using a cylindrical instrument (e.g., punch) and goniometer measurement relative to the scalp surface. This provides a practical and reproducible approach for clinical estimation.

Importantly, this framework is not based on density alone, but integrates multiple determinants of visual coverage, including hair diameter, length, and exit angle. The presented clinical examples are intended to illustrate model behavior rather than provide definitive validation.

Additional factors, such as hair–scalp color contrast and postoperative hair styling, may further influence perceived density. Despite these limitations, this work represents an initial step toward a quantitative, geometry‐based approach to donor evaluation and individualized surgical planning. Future prospective studies correlating predicted and observed donor thinning will be important for validation.

Furthermore, the proposed framework is intended to be applied on a region‐by‐region basis rather than relying on a single average value for the entire donor area. By incorporating local hair density, hair caliber, and exit angle, the model can guide individualized harvesting across anatomically distinct donor subregions, including the occipital, parietal, and temporal regions, while preserving a visually homogeneous donor appearance. This regional approach recognizes that the perception of donor thinning is primarily influenced by the visual contrast between adjacent donor subregions rather than by the global average donor density. Accordingly, each donor subregion should remain above its calculated minimum geometric coverage threshold, thereby minimizing abrupt transitions between harvested and adjacent non‐harvested areas. This hypothesis represents one of the principal predictions of the proposed framework and warrants prospective clinical validation.

This concept is particularly relevant in patients wearing short hairstyles, where geometric projection predominates and the model is expected to provide its greatest predictive accuracy. Accordingly, harvesting should be distributed in a graded manner, with progressively lower extraction densities toward the peripheral borders of the harvested area to create a smooth transition between treated and untreated regions while remaining within the anatomical limits of the safe donor area. In patients approaching the maximum safe harvest, individualized surgical planning may be required to balance donor safety with aesthetic homogeneity across the occipital, parietal, and temporal donor subregions.

Importantly, clinically significant visual contrast is primarily a concern in patients wearing short hairstyles, which also represents the clinical scenario in which the proposed framework is expected to provide its highest predictive accuracy. Nevertheless, the present model remains a theoretical geometric framework and has not yet been prospectively validated using clinical outcome data. Therefore, it should be applied with appropriate clinical judgment, and further prospective clinical studies are warranted to verify its predictive accuracy, determine its clinical utility, and validate its performance under different donor characteristics, hairstyles, and harvesting patterns.

### 4.1 Clinical Implications

In clinical practice, this geometric model may assist the surgeon in individualizing donor harvesting strategies. Before extraction, patient‐specific parameters—including hair length, hair diameter, exit angle, and donor density—can be incorporated to estimate theoretically safe harvesting limits. During consultation, the model may help illustrate how variations in hair geometry influence visual donor coverage after extraction. Postoperatively, it may also provide guidance regarding hair length and styling to optimize perceived donor density. Overall, the model represents a practical framework for individualized donor management and may contribute to reducing the risk of overharvesting.

Specifically, this approach may assist in: (1) Avoiding overharvesting by offering approximate quantitative estimates of safe extraction limits. (2) Supporting objective donor assessment, complementing empirical evaluation. (3) Improving planning and patient communication, by clarifying expected donor appearance and extraction capacity based on individual hair characteristics. (4) Guiding postoperative hair styling, which can influence perceived donor density.

## 5 Conclusion

This work proposes a simplified geometric approach for estimating donor coverage and the risk of visible thinning after FUE. By integrating hair length, diameter, exit angle, and density, it provides a structured framework for understanding visual density beyond hair density alone.

The model represents a first‐order approximation of a complex physiological system and is intended to provide clinically useful estimates rather than exact predictions. Its accuracy is inherently limited by variability in hair morphology, optical effects, and scalp characteristics.

Despite these limitations, the approach establishes a quantitative relationship between key parameters that influence donor appearance and are often considered qualitatively in practice. It suggests that visual coverage depends on multiple interacting factors and that safe extraction limits are individualized rather than universal.

Further studies are required to validate the model, refine its parameters, and assess its clinical applicability.

## Author Contributions

Conception of the manuscript was done by G.Z. and A.K.G. The manuscript was drafted by G.Z., V.E., and M.T. The manuscript was substantively edited and revised by A.K.G., G.Z., P.T.R., and M.T.

## Funding

The authors have nothing to report.

## Ethics Statement

The authors have nothing to report.

## Consent

Written informed consent was obtained from the patient(s) for publication of the clinical photographs and any accompanying clinical information.

## Conflicts of Interest

The authors declare no conflicts of interest.

## Data Availability

Data can be made available upon reasonable request.
